# The nephron arterial network and its interactions: nonlinear and information theoretic analyses

**DOI:** 10.3389/fnetp.2026.1842320

**Published:** 2026-09-11

**Authors:** Donald J. Marsh, Niels-Henrik Holstein-Rathlou

**Affiliations:** 1 Department of Medical Sciences, Division of Medicine and Biological Sciences, Brown University, Providence, RI, United States; 2 Department of Biomedical Sciences, Panum Institute, University of Copenhagen, Copenhagen, Denmark

**Keywords:** arterial network, autoregulation, chaos, kidney, mutual information, network physiology, oscillation, renal blood flow

## Abstract

Nephrons receive blood from a tree-shaped network of arteries and arterioles and regulate demand by actions that affect the diameters of their afferent arterioles. The network structure is asymmetric. The flow of blood requires a gradient of hydrostatic pressure so that the blood pressure at the origins of different afferent arterioles must differ from each other. Nephron mechanisms that act on afferent arterioles include a myogenic mechanism, a common feature of smooth muscle cells throughout the body, and tubuloglomerular feedback (TGF), a negative feedback mechanism that senses concentrations of common electrolytes in tubular fluid as it passes from the thick ascending limb into the distal tubule. The myogenic mechanism generates an autonomous oscillation in the electrical potential difference of the plasma membrane. The TGF signal also oscillates, albeit at a lower frequency, and modulates the frequency and amplitude of the myogenic mechanism’s potential difference oscillation. The interaction of TGF with the myogenic mechanism generates a bimodal electrical signal that enters the adjacent arterial segment. The arterial segments of the network are electrically conductive, providing a channel for nephrons to interact with each other. In addition, the oscillation of nephron blood flow caused by the myogenic-TGF interaction leads to changes in local arterial blood pressure, providing a hemodynamic interaction among nephrons. We developed a mathematical model that incorporates these features. The model demonstrates renal autoregulation and the oscillations in tubular fluid flow and pressure found in experiments. The results illustrate the effects of interactions on the network. In this paper we estimate Lyapunov exponents to establish the dynamic state of each nephron, and mutual information to provide quantitative evaluations of the importance of each of the information streams on nephron dynamics. The results indicate that all nephrons operate in a state of weak chaotic dynamics and that electrical signaling through the arteries exerts a greater influence on nephron dynamics than do hemodynamic interactions. Chaotic attractors are sensitive to initial conditions, and we suggest that the nephron cluster is therefore sensitive to the information streams in the system, enabling the cluster to adapt to changing external conditions.

## Introduction

Nephrons regulate blood flow by adjusting the diameters of their afferent arterioles when blood pressure changes. A myogenic mechanism, similar to those found in arterioles in other organs, generates autonomous oscillations in the arteriolar diameter because of interactions among permeabilities of the plasma membrane, leading to oscillations in intracellular ${\text{Ca}}^{2+}$ concentration, which in turn cause oscillations in the lengths of sliding muscle filaments. A kidney specific mechanism, tubuloglomerular feedback (TGF), originates in the macula densa, a group of 35–40 epithelial cells at the junction of the thick ascending limb of Henle’s loop and the early distal tubule. Macula densa cells sense the concentration of common monovalent ions in the tubular fluid, and generate a signal that is conducted to smooth muscle cells of the nephron’s afferent arteriole, where it affects the intracellular ${\text{Ca}}^{2+}$ concentration. The mechanism generating the TGF signal is non-linear, and it causes an oscillation of afferent arteriolar diameter whose frequency is dependent on the length of the tubule from its origin at the glomerulus to the macula densa, and lower than the frequency of the myogenic mechanism. Because both mechanisms act on ionic permeabilities affecting the intracellular ${\text{Ca}}^{2+}$ concentration they interact, the interaction leads to synchronization of the two rhythms at integer ratios of myogenic/TGF frequencies of 4, 5, or 6, with TGF modulating both the amplitude and frequency of the myogenic oscillation (Marsh et al., 2005a; Marsh et al., 2005b; Sosnovtseva et al., 2007).

The TGF mechanism forms the basis for a negative feedback mechanism that regulates nephron blood flow, glomerular filtration, and tubular flow. Because of its location, the macula densa sensor serves to constrain the mass load of electrolytes delivered to downstream epithelial transport mechanisms responsible for regulating electrolyte excretion (e.g., ${\text{Na}}^{+}$ , ${\text{K}}^{+}$ and ${\text{Cl}}^{-}$ ). These transporters respond to signals from extra-renal sensors and serve to regulate extracellular fluid volume, but their finite capacity requires regulation of the mass load presented to them. Many of the epithelial transport mechanisms in the proximal tubule and loops of Henle are flow rate dependent, and TGF maintains appropriate flow rate levels throughout the nephron (Edwards et al., 2025). This formulation for a single nephron, in a model with representations of epithelial transport processes, predicted tubular pressure fluctuations and autoregulation, but lacked components of the arterial network that supplies blood to the afferent arterioles (Marsh et al., 2017; Marsh et al., 2009; Laugesen et al., 2010).

The arteries that make up the network have structural and functional properties that suggest a role in the dynamics of the network. First, for blood to flow from the renal artery through the network to the renal vein requires a gradient of hydrostatic pressure. The existence of such a gradient means that afferent arterioles whose origin sites differ will experience different vascular pressures. Second, the lengths of arterial segments separating adjacent afferent arterioles follow a Poisson distribution (Marsh et al., 2017; Postnov et al., 2016). Such a distribution introduces randomness and implies an asymmetry in the vascular network. Third, we identified three patterns in the origins of afferent arterioles: 53% in clusters of two or more arterioles at the terminal ends of the network at the renal surface; 23% as arteriolar pairs from unpaired sub-surface arteries; and 24% as single arterioles from sub-surface arteries (Marsh et al., 2017). Clusters conforming to these distributions also break symmetry. Finally, arterial branches of the renal artery run parallel to the renal surface but do not give rise to afferent arterioles. Branches arise at intervals from these parallel arteries and run orthogonally toward the renal surface. These orthogonal arteries form as few as two or as many as seven branches before reaching the renal surface, and provide the sites of origins for afferent arterioles. The variability in numbers of branches also breaks symmetry. Broken symmetry often gives rise to emergent properties (Krakauer, 2023).

In our numerical experiments on the nephron arterial network we used a constant blood pressure at the root of the tree network (Marsh et al., 2023). The afferent arteriole closest to the root oscillates and withdraws blood periodically, imposing periodicity on blood flow to the remaining downstream arterial segments. This process repeats at each branch point, and there are periodic changes in blood pressure at all branch points. The periodicity approximates the TGF frequency. These fluctuations in vascular pressure constitute an information stream.

Each afferent arteriole, as described above, has a myogenic mechanism that generates oscillations in the membrane potential that affect intracellular ${\text{Ca}}^{2+}$ concentrations. The TGF signal from the macula densa modulates both the amplitude and the frequency of the myogenic oscillations, generating a bimodal electrical signal that is conducted to adjacent endothelial cells of the arterial segment. The endothelium is electrically conductive, and adjacent afferent arterioles exchange electrical signals, forming an information stream. Experimental verification of this formulation was provided by varying tubular perfusion rates past a single macula densa in a cluster of arteries and afferent arterioles and loaded with a voltage sensitive dye. An increase in tubular perfusion evoked a change in membrane potential in the perfused nephron; the propagation of this change to attached neighbor afferent arterioles, and the decline in membrane potential changed with arterial distance from the stimulated nephron (Marsh et al., 2009). These experimental results show that TGF generates an electrical signal, that the signal is propagated into connected arteries and arterioles, and that the propagation is electrotonic, i.e., it occurs through an electrically resistive network not involving regenerative action potential propagation. Thus, the TGF signal to the afferent arteriole constitutes a third information stream.

This is the third in a series of papers that simulate a cluster of nephrons and the arteries that supply them with blood to address the dynamics of the nephron arterial network. The first paper presented the model and analyzed the interactions among the nephrons (Marsh et al., 2023), all at constant resting-level arterial blood pressure. A random number generator was used to vary the lengths of arteries and nephrons in 15 simulations. The second paper used one of the 15 simulations, and, starting at resting-level arterial pressure applied an acute increase of arterial pressure (Marsh and Holstein-Rathlou, 2024). Separate simulations were made at seven different blood pressure levels in the interval [85,115] mmHg, to simulate the response to acute hypertension. The analysis concentrated on the tubular response to the blood pressure change.

In this paper we analyze the various information streams generated by the model and their responses to acute hypertension. Much of the analysis of nephron dynamics, both experimental and simulated, has provided calculations of the power spectrum derived from the application of Fourier transforms. The nephrons and the network are non-linear but the Fourier transform is a linear operation. The analysis in this paper applies analytic methods designed for non-linear systems. One of these methods estimates Lyapunov exponents to determine whether the fluctuations in each process operate as limit cycle or quasi-periodic oscillators, or as chaotic attractors. Phase space plots from a small number of nephrons were consistent with chaotic dynamics (Marsh and Holstein-Rathlou, 2024). One distinction between these states has to do with the sensitivity to initial conditions. Chaotic dynamics are sensitive to initial conditions, a sensitivity that would lend itself to participation in information exchange among nephrons, while limit cycle and quasi-periodic oscillators do not show sensitivity to initial conditions. We have therefore applied the Lyapunov exponent measure to each of the 10 nephrons in the model, and at seven different levels of application of acute hypertension, to determine the generality of the chaotic state. The information streams operating in this system are physical variables, each with its own dimensions. Comparisons of the strength of the information streams require a transformation of the variables to time series with a common dimension. We use mutual information, a procedure derived from Shannon’s information theory (Shannon and Weaver, 1998), for this purpose.

## Methods

**FIGURE 1:**
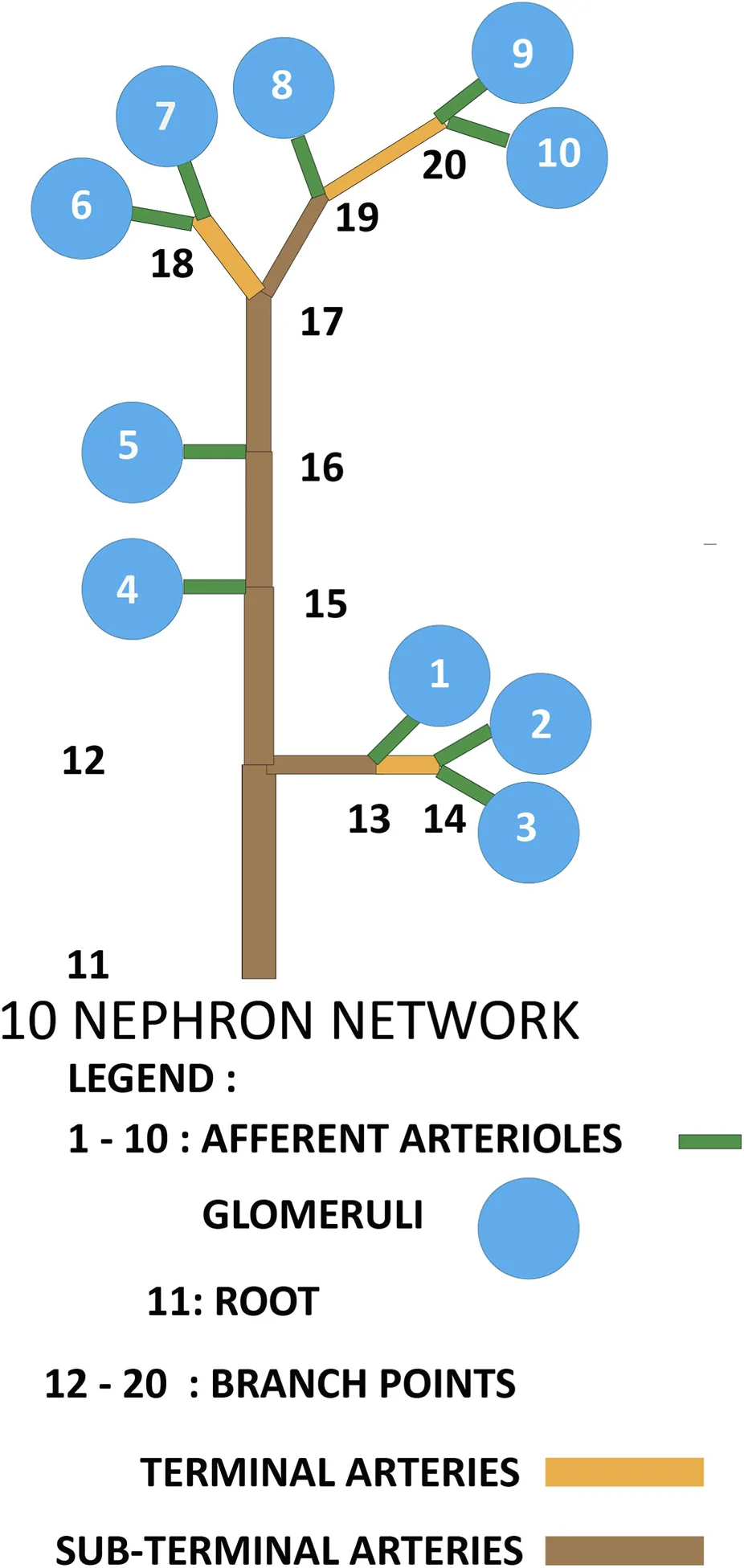
Diagram of the nephron-arterial network used for simulations. Arterial lengths and diameters are drawn to an identical scale. Arterial lengths were provided as initial conditions, arterial diameters were calculated using a relaxation method, and both were held constant during the acute hypertension simulations. Afferent arteriolar lengths are not drawn to scale, all were assigned the same lengths in the simulations, and the arteriolar diameters varied during the course of the acute hypertension simulations.

**FIGURE 2:**
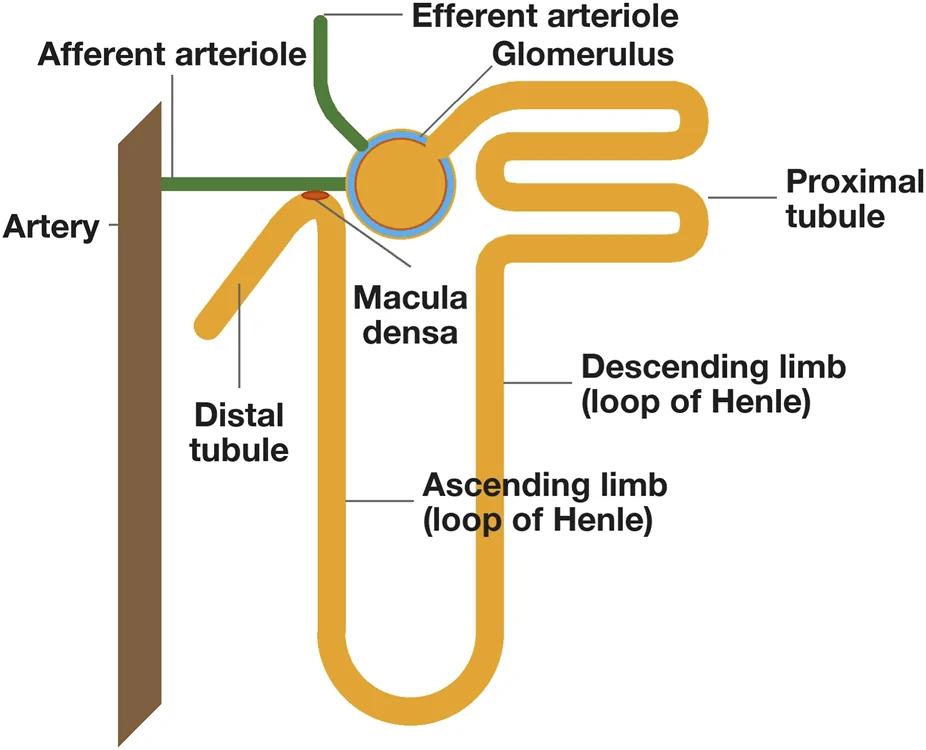
Diagram of an artery, arterioles, glomerulus, tubule, and the macula densa. The figure applies to each of the nephrons in the cluster. The 10 models vary from each other only in their lengths.

The simulations in this study are based on a model containing 10 single nephron models, each supplied by a single afferent arteriole originating from an arterial branch (Marsh et al., 2023). Figure 1 shows the overall model structure, Figure 2 more detailed illustration of the nephron, its segments, the macula densa, and the arterial connections. The equations of the single nephron-afferent arteriolar model and the network are in the Supplementary Material attached to this manuscript.

The nephron model used in each of the 10 nephrons was first developed to simulate the dynamics of a single nephron (Marsh et al., 2005a; b), and later expanded to simulate two interacting nephrons (Marsh et al., 2009). TGF gain was then shown to serve as a bifurcation parameter for these models (Laugesen et al., 2010), capable of eliciting quasi-periodicity, limit cycle oscillations, or deterministic chaos while the blood pressure was held constant. This model was used for each of the 10 nephron sites in the network model, except that TGF gain was held constant while blood pressure was varied.

For each of the 10 nephron models glomerular filtration rate is calculated with an ordinary differential equation for plasma protein concentration as a function of distance along an idealized glomerular capillary. Glomerular filtration rate provides an initial flow condition for the tubular model, a set of partial differential equations that calculates hydrostatic pressure, tubular fluid flow, and NaCl concentration as functions of time and distance along the nephron. The nephron is modeled as a compliant tubule of epithelial cells that transport water and solutes between the lumen and the interstitial fluid. The nephron model has three segments: proximal tubule, descending limb of Henle’s loop, and thick ascending limb of Henle’s loop. Each segment is provided with epithelial transport functions and compliance values determined from published experimental values. The terminal tubular flow rate and NaCl concentrations result from the epithelial transport processes; the terminal hydrostatic pressure is calculated from a non-linear function of the terminal fluid flow. The system of partial differential equations describing tubular pressures, flows, and concentrations and the system of ordinary differential equations describing afferent arteriolar activities are solved iteratively at each time step.

Afferent arterioles were simulated using a set of 6 ordinary differential equations developed by Gonzalez-Fernandez and Ermentrout to predict blood flow dynamics in cerebral arteries (Gonzalez-Fernandez and Ermentrout, 1994). This model simulates voltage gated ${\text{Ca}}^{2+}$ channels and voltage- and ${\text{Ca}}^{2+}$ -sensitive ${\text{K}}^{+}$ channels in smooth muscle cells. The interacting ionic currents are non-linear and produce autonomous oscillations of the membrane electrical potential difference and of intracellular ${\text{Ca}}^{2+}$ . The ${\text{Ca}}^{2+}$ oscillation induces oscillations in myosin light chain phosphorylation, cross bridge formation, and muscle stress. The model simulates hoop, elastic, and muscle stresses, and from them, the rate of change of the arteriole’s diameter.

The Gonzalez-Fernandez-Ermentrout model (Gonzalez-Fernandez and Ermentrout, 1994) simulates the action of the myogenic mechanism in arterioles throughout the body and is not specific to the kidney. TGF is a renal-specific mechanism whose signal originates in the macula densa and is transmitted through the mesangium to the afferent arteriole. Our model couples the TGF signal to the membrane ${\text{Ca}}^{2+}$ permeability of the arteriolar smooth muscle cell. The TGF signal arrives at the afferent arteriole at a site near the nephron’s glomerulus and its action propagates upstream. To simulate this non-uniform distribution we modeled the afferent arteriole as 2 separate segments in series coupled electrically, the segment closer to the glomerulus receiving the larger TGF signal.

We solved the 10 nephron model assuming conservation of blood flow and electrical currents at each vascular node. We also assumed that the combination of TGF and the myogenic mechanism acting on the afferent arteriole were responsible for all adjustments to arteriolar diameter in each nephron with no active responsive elements in the arteries. Lengths of arterial segments were based on measurements made from a vascular cast of a rat kidney (Marsh et al., 2017). Because optical methods for measuring vascular radii lack sufficient resolution we used a relaxation method to estimate arterial radii at a resting arterial blood pressure (Marsh et al., 2023; Marsh and Holstein-Rathlou, 2024). Upon completion of the relaxation protocol, we produced acute hypertension with an hyperbolic tangent function applied to node 11 blood pressure.

In the initial presentation of the model (Marsh et al., 2023) we used a random number generator to vary the lengths of nephrons and arterial segments in 15 simulations, each simulation using a unique seed value. This strategy was designed to evaluate the effects of geometric variation on synchronization among the information streams. For the study of the effects of acute hypertension we selected a single one of the 15 simulations and applied 7 different levels of arterial pressure at Node 11 (Marsh and Holstein-Rathlou, 2024).

We first tested the network model for its ability to simulate autoregulation of renal blood flow. The initial formulation failed this test, but succeeded with two modifications. The functions that simulate pressure diuresis, a known inhibition of proximal tubule fluid reabsorption by increased blood pressure (Chou and Marsh, 1988; 1998), and the inverse relationship between efferent arteriolar vascular resistance and arterial blood pressure (Sorensen et al., 2000; Sorensen et al., 2004). These modifications are included in each of the 10 nephron models.

All simulations were begun with the blood pressure at Node 11, the root of the arterial tree, set to 85 mmHg. Initial simulations ran for 1,000 s at this resting blood pressure level after which acute hypertension was induced. These simulations ran for 4,000 simulated seconds, with a time interval between data points of 0.25 s, corresponding to a total of 16,000 data points in each run. Presentation of the results and data analysis were begun at 1,000 simulated seconds, allowing the first 1,000 s to serve as a period of relaxation from the initial increase in arterial pressure. We exercised the model at 7 different values of Node 11 pressure: 85, 90, 95, 100, 105, 110, and 115 mmHg.

In some of the simulations we used a blood pressure input with a *1/f* noise pattern. The *1/f* time series were created using the routine dsp. ColoredNoise in Matlab (R2023a). The results were plotted using wavelet transforms with the default analytic Morse wavelet, also from Matlab. Wavelet transforms are 3-dimensional plots showing spectral power as a function of time and frequency. The intent in these simulation was to use a blood pressure forcing with the same statistical properties as those found in unrestrained conscious rats with implanted units containing arterial catheters, pressure transducers, and radiotransmitters (Holstein-Rathlou et al., 1995). To be more precise, the general formula for the pattern is $1/f^{\alpha }$ . The measured value of α was 1.45, and this value was used to generate the blood pressure time series used in the simulations.

Numerical methods are described in (Marsh et al., 2023; Marsh and Holstein-Rathlou, 2024).

### Data analysis

One goal of this study was to determine whether the predicted nephron time series could be characterized as limit cycle oscillations or deterministic chaos. The dynamics of a system depends on its sensitivity to initial conditions: limit cycle oscillators are insensitive to initial conditions and will return to their original trajectories when perturbed, while chaotic systems are sensitive to initial conditions and successive trajectories will diverge exponentially, giving rise to apparently random dynamics. In the context of a model of interacting nephrons the interacting information streams function as perturbations. Measurement of Lyapunov exponents has become standard in determining the nature of the dynamics (Pikovsky et al., 2001). Lyapunov exponents measure the rate at which nearby trajectories converge or diverge with time. Limit cycles have negative Lyapunov exponents, chaotic systems positive exponents. The magnitude of the exponent expresses the rate of convergence or divergence. To determine whether the nephron time series were characteristic of limit cycle oscillations or of deterministic chaos, we calculated the largest Lyapunov exponents for each of 10 nephrons at each of seven node 11 pressures from the last 8,000 simulated seconds recorded at 0.25 s intervals. The Lyapunov exponents were estimated using a Python3 program containing the nolds library. Mutual information was estimated using a Python3 program containing the sklearn. feature_selection of the scikit_learn library.

To confirm that the Lyapunov exponent estimates did, in fact, identify chaotic dynamics, the response of the system to a small perturbation of the tubular pressure ( $10^{-3}$ mmHg) was determined. The system response to the perturbation was determined by estimating the local Jacobian at each time point, and using this to predict the new state of the perturbed system at each future time point (Eckmann and Ruelle, 1985). Chaotic dynamics is characterized by divergence of trajectories in phase space, and over time a perturbation grows to equal the size of the attractor. The time to reach this level is denoted the prediction horizon, ${\text{t}}_{\mathrm{pred}}$ , and is a measure of the degree of chaos in the system (Eckmann et al., 1986; Sano and Sawada, 1985). More chaotic conditions reach ${\text{t}}_{\mathrm{pred}}$ sooner, weakly chaotic ones later.

Another goal was to compare the influence of information streams conducted through arteries - electrical and hydrodynamic - on autonomous processes generated within each nephron. For linear systems this comparison could be made with correlation coefficients, but the nephron arterial network is distinctly non-linear. A further complication is that the interactions of interest are physical processes, each with its own dimensions. But each physical process also has information content, and the comparisons can be made using mutual information, a procedure derived from Shannon’s information theory (Shannon and Weaver, 1998), that measures the statistical dependence between two variables by comparing their probability density functions. Mutual information can detect both linear and non-linear dependencies and expresses the results in bits. To compare the effects of each information stream - TGF, arterial electrical current flow, and blood flow in each arterial branch - we applied the method of continuous mutual information for each of 7 node 11 pressures from the last 8,000 simulated seconds recorded at 0.25 s intervals.

## Results

Experimental results used for model validation came from a variety of sources: tubular pressure measurements at basal blood pressure, (Holstein-Rathlou, 1987; Holstein-Rathlou and Leyssac, 1986; Holstein-Rathlou and Marsh, 1989; Yip et al., 1992; Yip et al., 1993). Doppler flow measurements from single efferent arterioles made at control and acutely elevated blood pressure (Yip et al., 1993), laser speckle contrast spectrometry at basal blood pressure (Brazhe et al., 2014; Postnov et al., 2022.), and tubular microperfusion at control and acutely elevated blood pressure (Chou and Marsh, 1998) all in anesthetized male rats. These results show synchronization of tubular pressure oscillations in tubules near each other (Holstein-Rathlou, 1987), and later specifically among nephrons arising from a single artery, but not between those arising from different arteries (Yip et al., 1992). Figures from these various reports of tubular pressure measurements invariably reveal, in addition to the oscillation attributed to TGF, slower and lower amplitude changes. These recordings were typically too short for Fourier based spectral analysis, but the longer periods of simulation in this study permit us to test whether these slower fluctuations are an emergent property of the system.

**FIGURE 3:**
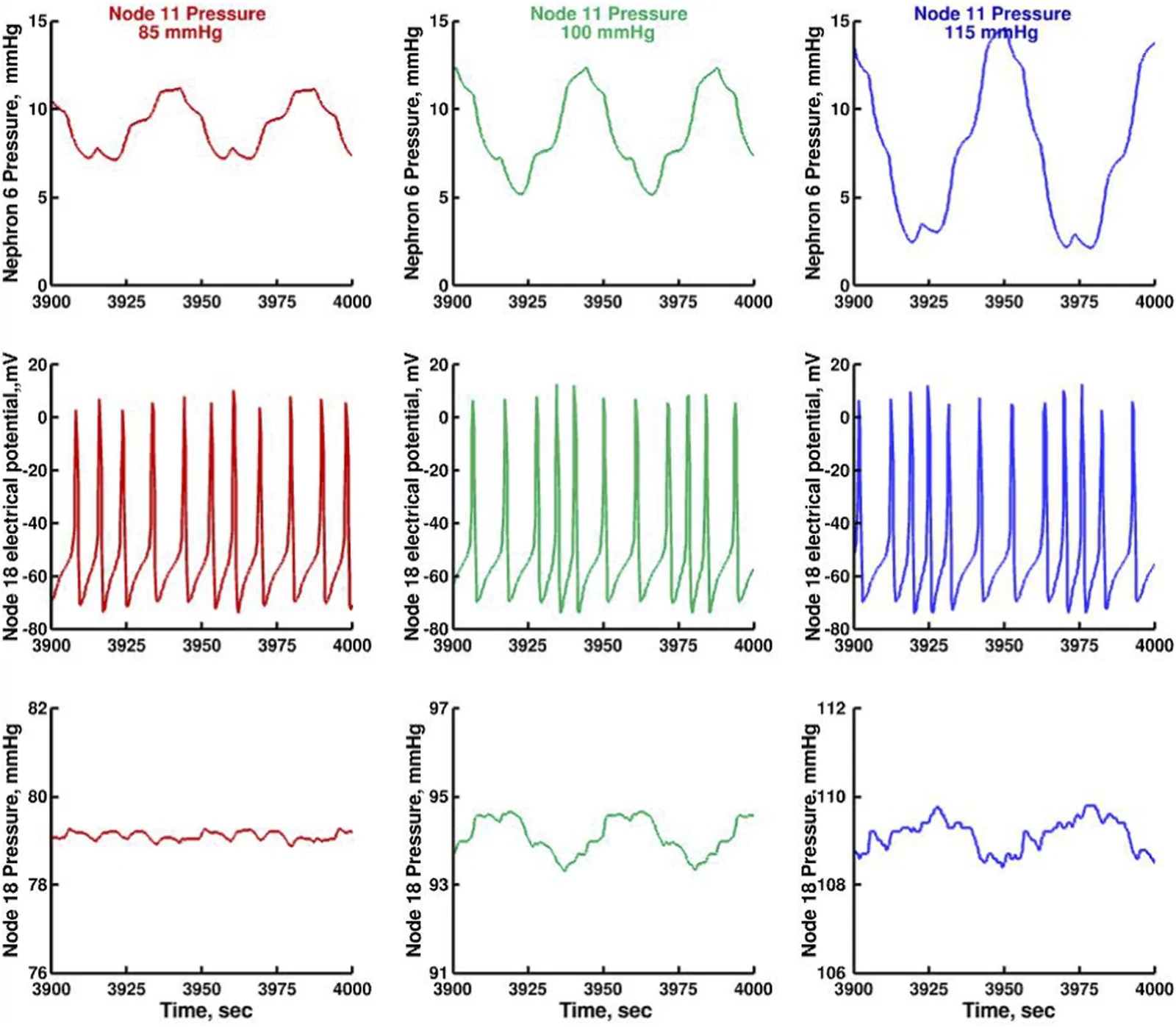
Time series of Nephron 6 proximal tubule pressure, Node 18 electrical PD, and Node 18 arterial pressure, at 3 different Node 11 arterial pressures. Nephron six is a surface nephron. Node 18 is at the origin of the afferent arteriole supplying blood to Nephron 6. The PD is in the endothelium at Node 18. Node 11 is the root of the 10 nephron set simulated in this study. Node 11 pressure of 85 mmHg, with results in Column 1, is the reference state for the 10 nephron set. Results in the 2nd and 3rd columns are shown after an increase in Node 11 pressure from 85 mmHg and a suitable period for the adapted nephrons to accommodate to the pressure change.

Figure 3 shows the proximal tubule hydrostatic pressure in Nephron 6, endothelial electrical potential in Node 18, and arterial nodal blood pressure in Node 18. Nephron six is a surface nephron, and Node 18 is the site of arterial origin of the afferent arteriole supplying Nephron 6. The figure shows the final 100 simulated seconds at each of three different node 11 arterial pressures. Both the tubule and arterial pressures record complex fluctuations with amplitudes that increase with increasing node 11 pressure. The larger component of each of these pressure records is the TGF oscillation, the smaller is the myogenic oscillation. The duration of each TGF oscillation increases with increasing Node 11 pressure. The middle row shows the electrical potential in the endothelium at Node 18. The major component of the electrical potential signal is the myogenic oscillation, and the largest effect of increasing Node 11 pressure is a phase shift between successive action potentials; amplitude variation, while present, is minor. In the development of the model (Marsh et al., 2005a; Marsh et al., 2005b), and confirmed experimentally (Sosnovtseva et al., 2007), we showed that TGF modulates both the amplitude and frequency of the myogenic oscillation, a result reflected in this figure. The predicted nodal electrical potential in Figure 3 also show phase angles that are both time and blood pressure dependent.

**FIGURE 4:**
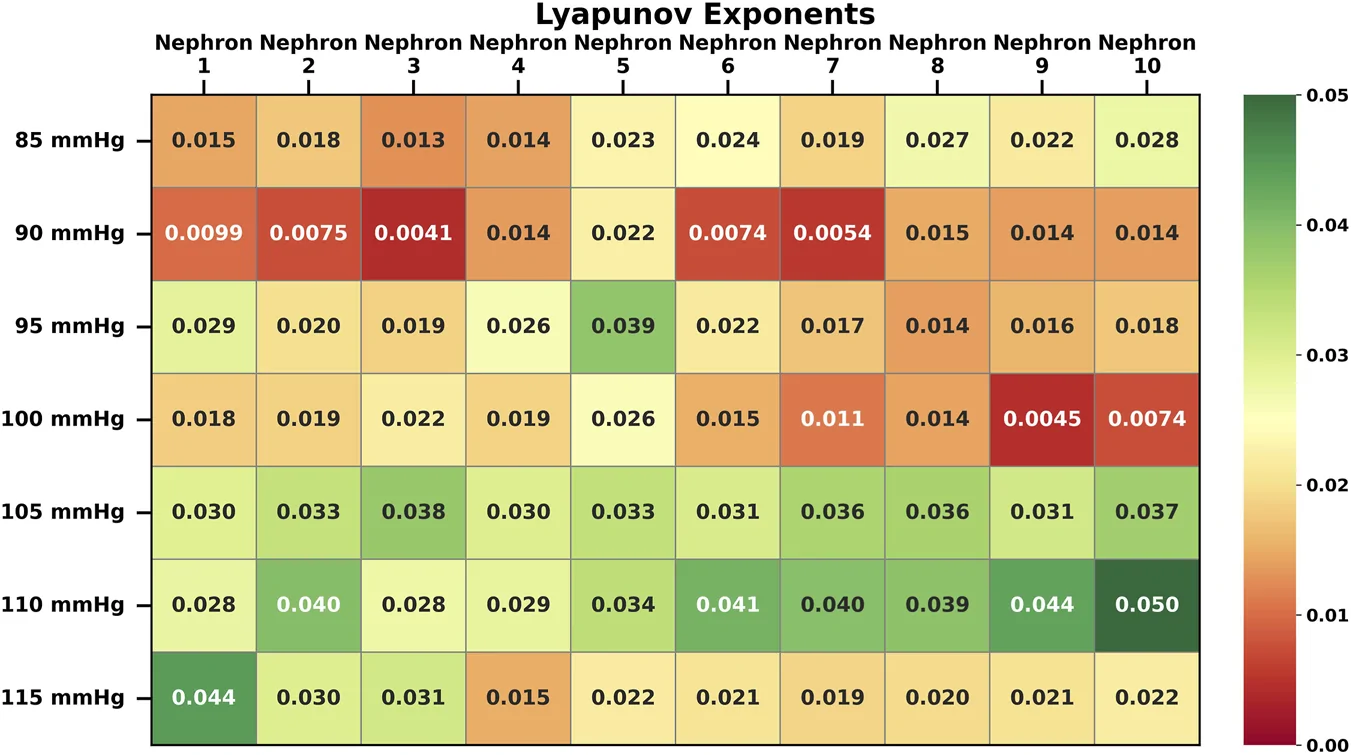
Lyapunov exponent results in each of 10 nephrons, after blood pressures of 90, 95, 100, 105, 110, and 115 mmHg had been applied acutely to Node 11. The simulation at 85 mmHg was the resting blood pressure state. Lyapunov exponents have the dimension of reciprocal time.

Figure 4 shows the Lyapunov exponents calculated from the proximal tubule time series from each of the 10 nephrons at the resting node 11 pressure, 85 mmHg, and at six additional Node 11 pressures after imposing a step increase in Node 11 pressure. Each of the simulations were run for 4,000 s and sampled at 0.25 s intervals. The increase in Node 11 pressure was applied at 1,000 s and the Lyapunov exponents were calculated from the simulations beginning at 2000 s and ending at 4,000 s. All 70 values were positive, indicating chaotic dynamics. The numerical values varied from tubule to tubule and from one Node 11 pressure to another, but in no distinct pattern.

**FIGURE 5:**
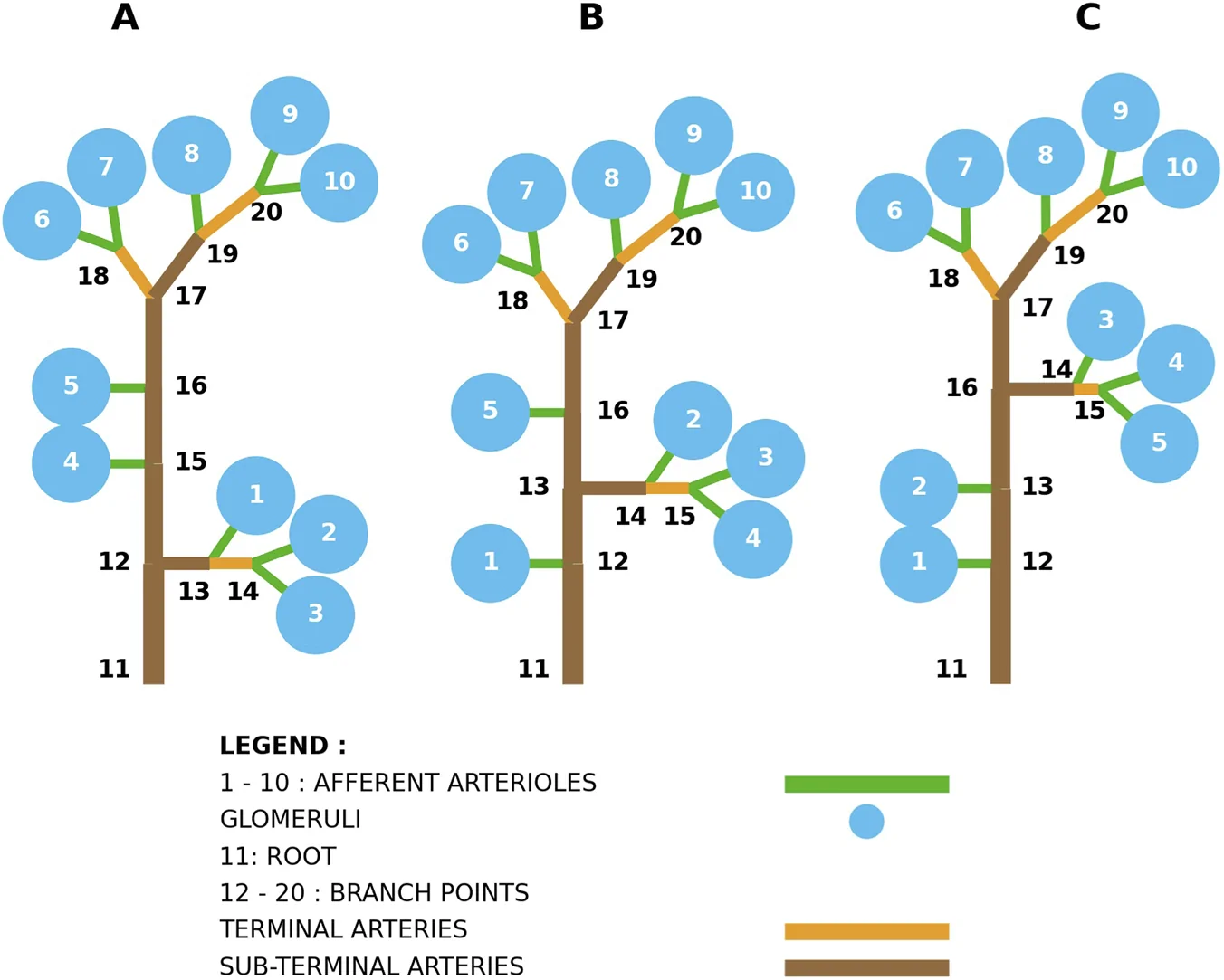
Three different network configurations used to test the generality of the nephron dynamics results. Topological variation was produced by altering the branch point of origin for the sub-surface cluster of three nephrons. Arterial lengths were also changed. Panel A is identical to Figure 1, and is included here to facilitate comparison of the three different configurations. The results from the analysis of the structures shown in Panels **(A–C)** are summarized in Table 1.

To test whether variations in the topology affected the values of Lyapunov exponents, we constructed 2 variations on the original design, varying the origin of the sub-surface 3 nephron cluster. The 2 variations are shown in Figure 5, the results in Table 1. Lyapunov exponents were estimated from 10 nephrons at 7 levels of root arterial blood pressure, in each of the three models. Lyapunov exponents were positive in all 210 nephrons. The exact values differed among the different topologies, but the average values and standard deviations were similar. These results provide support for the conclusion that the topological variation does not affect the conclusion that the interacting information streams induce the chaotic dynamics of this model. The autoregulatory indices for Models A, B, and C were −0.03, −0.03, and −0.05, respectively, showing that the autoregulatory function was not disturbed by the topological variation. In the following, we have therefore only used the initial topology (Model A) for the network.

**TABLE 1:** Lyapunov exponent statistics. All values ×10^−3^.

​	Model A		Model B		Model C	
	Mean	SD	Mean	SD	Mean	SD
Overall						
Overall	23.4	10.5	15.8	11.1	22.6	11.3
Min	4.2	​	0.6	​	0.1	​
Max	49.8	​	50.7	​	47.0	​
By blood pressure (mmHg)						
85	20.2	5.4	10.5	5.3	18.4	1.9
90	11.3	5.5	6.4	5.4	14.0	3.8
95	21.9	7.5	6.7	3.4	6.4	4.9
100	15.6	6.5	11.5	6.3	31.2	8.6
105	33.5	3.0	18.3	9.0	29.6	3.5
110	37.1	7.4	25.8	5.9	36.1	4.6
115	24.5	8.6	31.6	10.0	22.7	10.6
By nephron number						
1	24.8	11.6	13.4	7.9	26.9	12.1
2	23.8	11.0	17.9	8.1	23.5	6.2
3	22.1	11.4	11.7	8.5	21.5	9.6
4	21.0	7.2	14.1	10.3	22.2	12.5
5	28.4	6.7	18.2	7.4	23.0	11.6
6	23.0	10.9	18.7	16.4	20.1	11.2
7	21.0	12.5	14.4	11.8	18.3	11.2
8	23.5	10.6	11.5	12.0	22.1	12.5
9	21.6	12.7	15.5	11.8	23.1	15.2
10	25.1	14.5	22.9	16.1	25.8	15.1

**FIGURE 6:**
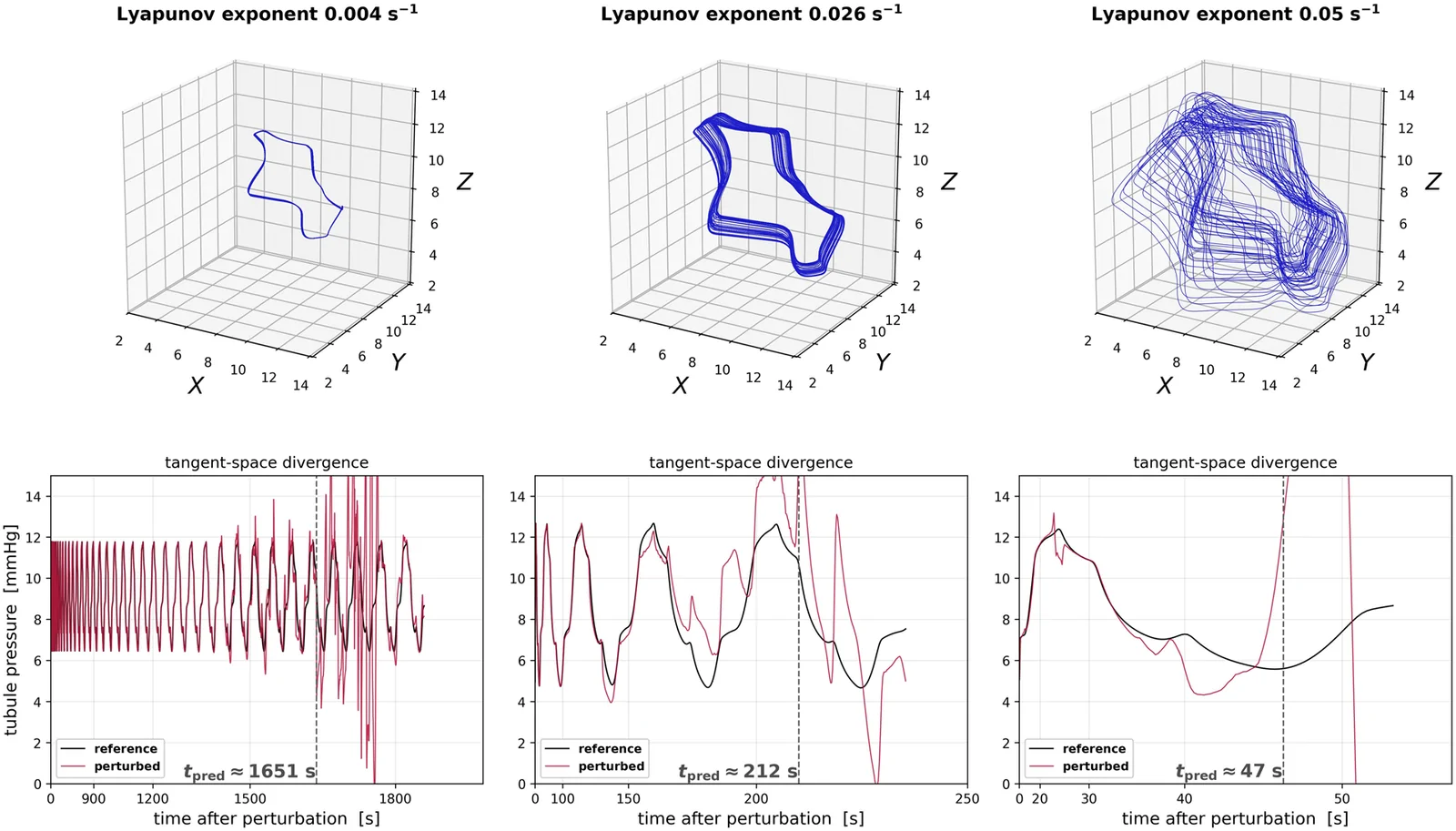
Top Row: Three dimensional phase space portraits created by plotting the original tubular pressure time series on the X-axis against 10 s and 20 s delayed versions of the same time series. X = tubular pressure, mmHg, Y = tubular pressure, mmHg, 10 s delayed from the original time series, and Z = tubular pressure, mmHg, 20 s delayed from the original time series. The figures show the lowest, midpoint, and highest Lyapunov exponents in Figure 4. These are Nephron 3 at Node 11 pressure of 90 mmHg, Nephron 5 at Node 11 pressure of 100 mmHg, and Nephron 10 at Node 11 pressure of 110 mmHg, respectively. Bottom Row: Tangent-space divergence for the corresponding attractor in the top row. The magnitude of the perturbation applied to each was $10^{-3}$ mmHg. ${\text{t}}_{\mathrm{pred}}$ is the time it takes for the perturbation to grow from $10^{-3}$ mmHg to the size of the attractor itself (the prediction horizon). The vertical dotted line indicates where the system reaches the prediction horizon. Delay-embedded phase portraits (top) and jacobian tangent-space perturbation growth (bottom). To better visualize the divergence of the trajectories the x-axes have been transformed using a third-degree polynomial.

In the top row of Figure 6 three dimensional phase portraits from Model A were produced by plotting tubular pressure time series against delayed versions of the same time series. The attractor with lowest Lyapunov exponent of the 70 is in the left panel, a portrait at the mid-level exponent in the middle panel, and a third portrait with the highest exponent at the right. The response of the system to a small perturbation of tubular pressure for the same group of attractors are shown in the bottom row. Initially, the perturbed and the reference trace are indistinguishable, but as time progresses they start to diverge. The left most attractor reached the prediction horizon at 1,651 s, the middle attractor at 212 s, and the right most attractor at 47 s. All three attractors diverged, indicating the presence of chaotic dynamics. The time to reach the prediction horizon was longest with the attractor with the lowest Lyapunov exponent, and shortest with the attractor with the highest Lyapunov exponent.

**FIGURE 7:**
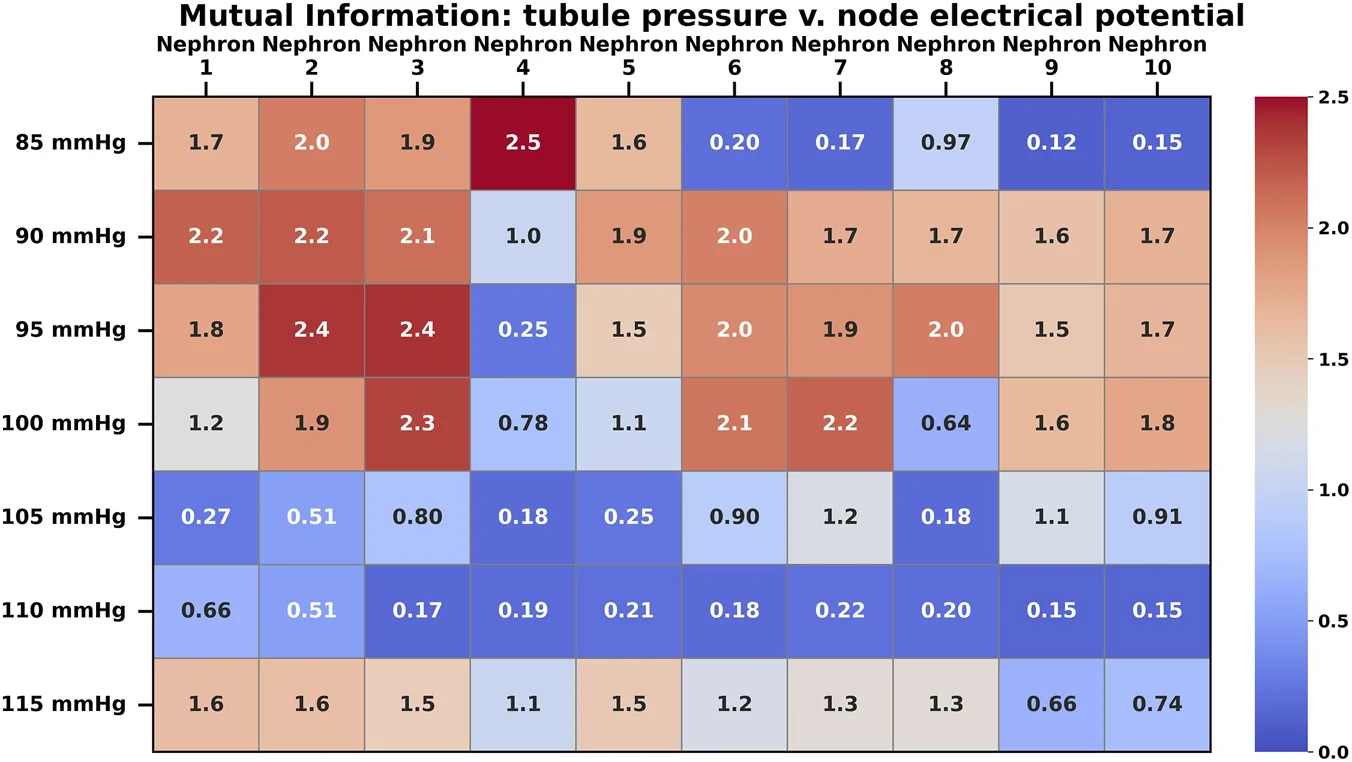
Mutual information between tubule pressure and arterial node electrical potential difference for each of 10 nephrons at node 11 pressures of 85, 90, 95, 100, 105, 110, and 115 mmHg.

Figure 7 presents the mutual information between tubule pressure and arterial node electrical potential for each nephron and the arterial node of origin of the nephron’s afferent arteriole, using the same simulation protocol described for Figure 3. As in Figure 3, the mutual information results varied from nephron to nephron and also among the node 11 pressures. The only consistent pattern occurred at node 11 pressure of 110 mmHg, where all nephrons exhibited low values.

**FIGURE 8:**
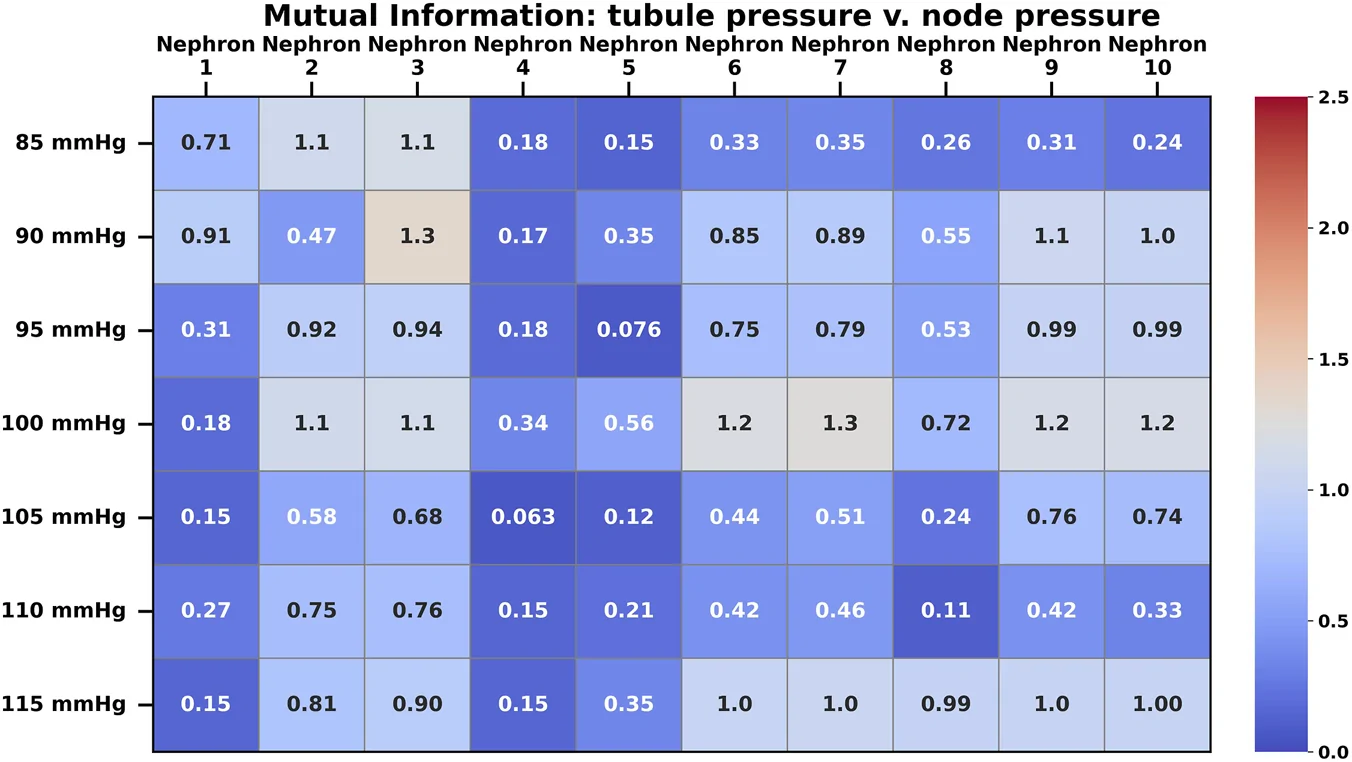
Mutual information between tubule pressure and arterial node vascular pressure for each of 10 nephrons at node 11 pressures of 85, 90, 95, 100, 105, 110, and 115 mmHg.

Figure 8 shows the mutual information of tubule pressure and vascular pressure at the arterial node of origin of each nephron’s afferent arteriole, using the same simulation protocol as in the preceding 2 figures. The color bar scale is the same as in Figure 7, but the values in this figure are lower. Node to node and node 11 pressure variations showed no consistent patterns.

Mutual information values with tubule pressure vs. node electrical potential were consistently higher than the values with tubule pressure vs. arterial node pressure, indicating that the variation in node PD was the more important determinant of the variation in tubular pressure.

**FIGURE 9:**
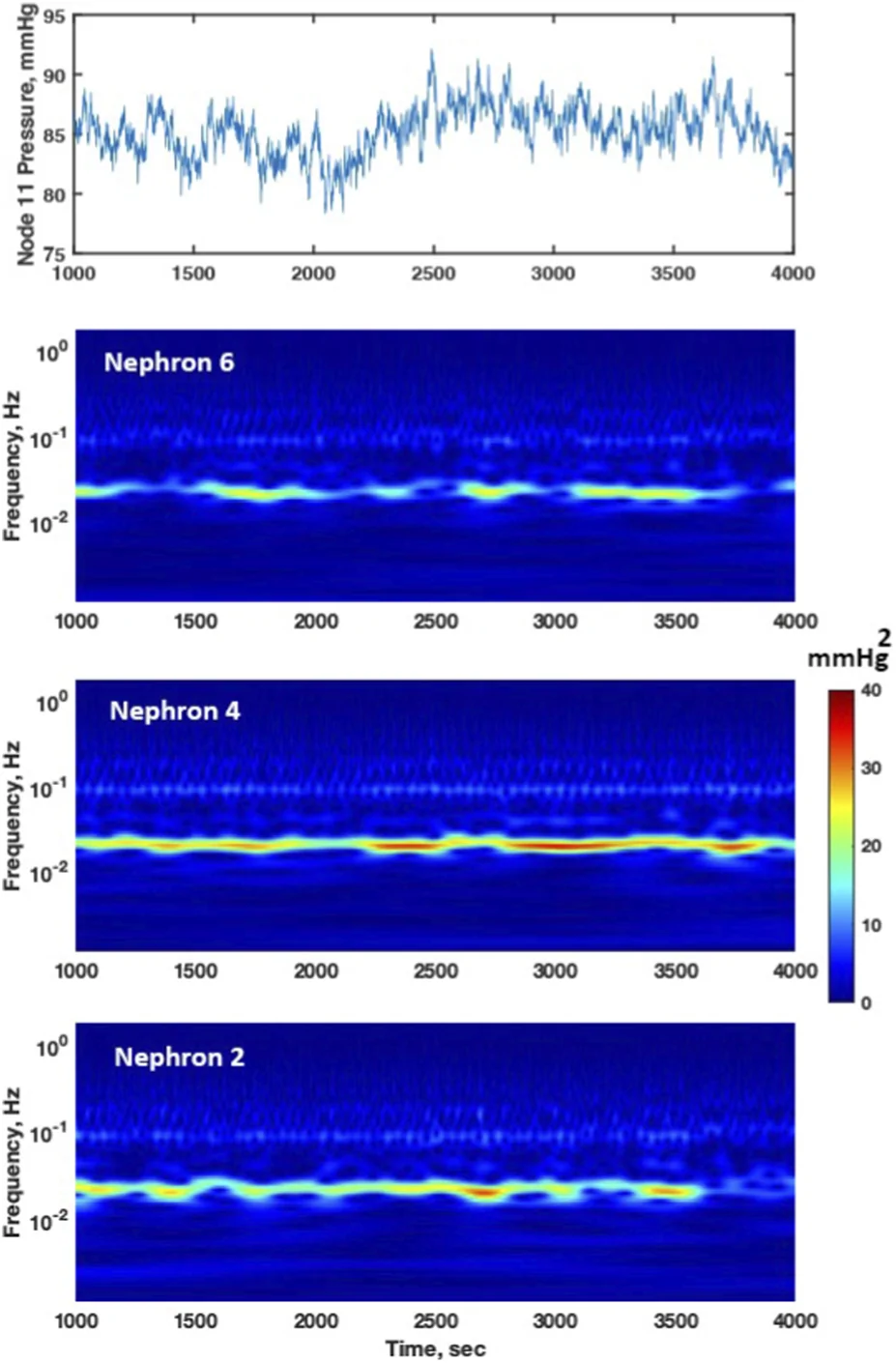
Wavelet transforms of Nephrons 2, 4, and 6 with an arterial pressure input of 1/f noise and mean pressure of 85 mmHg, shown in the top panel.

**FIGURE 10:**
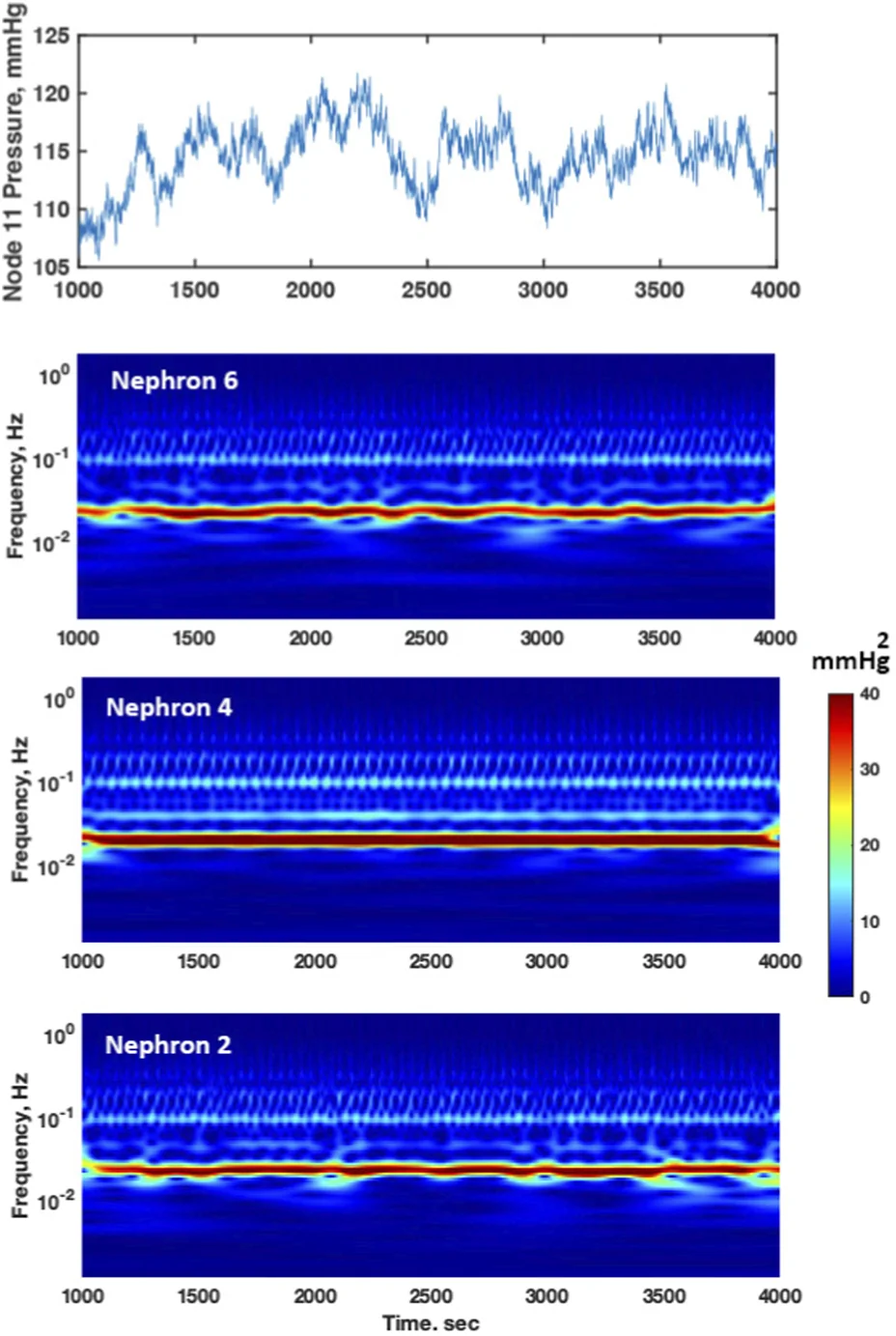
Wavelet transforms of Nephrons 2, 4, and 6 with an arterial pressure input of 1/f noise and mean pressure of 115 mmHg, shown in the top panel.

The results presented thus far were from simulations in which arterial blood pressure was held constant over time and then changed to another level, following which they were held constant over time. Blood pressure in conscious animals does not remain constant with time (Holstein-Rathlou et al., 1995; Marsh et al., 1990; Blinowska and Marsh, 1985; Wagner and Persson, 1994). For epochs of approximately 3 h the arterial pressure varies because of changes in activity; the pattern of blood pressure change has *1/f* statistics (Holstein-Rathlou et al., 1995; Marsh et al., 1990; Wagner and Persson, 1994). Figures 9, 10 show wavelet transform results from three nephrons during a *1/f* forcing of Node 11 pressure at mean arterial pressures of 85 and 115 mmHg. Nephron two is a member of a sub-surface nephron pair, Nephron 4 is an unpaired nephron arising from a sub-surface artery, and Nephron six is a member of a surface nephron pair. The results show a consistent maximum at the TGF frequency, and suggest that the system described in our model serves as a clock for the cluster of nephrons. The blood flow for the cluster during the *1/f* forcing was 2.63 nL/min with the mean blood pressure at 85 mmHg and 2.62 nL/min with the mean blood pressure at 115 mmHg.The autoregulatory index calculated from these results is −0.01, a value consistent with autoregulation despite the time varying root pressure.

## Discussion

Nephrogenesis, a process that also establishes the supporting vascular topology, is complete during gestation. Renal growth, as it progresses after birth, will change the lengths of arterial segments, but must retain the topology because new nephrons are not formed after gestation. Over the lifetime of the organism the blood pressure will vary, sometimes randomly and at other times periodically. Through all of these variations the kidneys must provide stability because physiologically appropriate tubular function requires close matching of tubular flow to epithelial transport capacity. In this sense the mechanisms responsible for autoregulation provide stability. We have developed a simulation that predicts the dynamics of tubular flow and pressure in a cluster of 10 nephrons connected to the arterial system that supplies blood to the nephrons. The topology of the arterial network is based on structural results from a micro-computerized tomographic study of a renal vascular cast (Marsh et al., 2017) and reflects the vascular asymmetries found there. The network is complex and therefore the interplay between structure and dynamics is important (Osipov et al., 2010). The tubular model was taken from previous studies (Marsh et al., 2005a; Marsh et al., 2005b, Marsh et al., 2009; Laugesen et al., 2010). That model, in one- and two-nephron versions, predicted limit cycle oscillations with the parameters used in this study. The afferent arteriolar component of the nephron model simulates the myogenic mechanism, activated by interactions among membrane ionic permeabilities, and, when modified by a signal from TGF, forms a bimodal electrical signal. The electrical transmission has been demonstrated in an experimental study of perfused nephron clusters (Wagner et al., 1997; Marsh et al., 2009). The electrical signal is conducted into a simulation of an endothelial component of the arterial wall, and propagated thence into the vascular network. The time-dependent change in arteriolar diameter causes a similarly time-dependent change in the blood flow to each nephron, and results in a time-dependent change in vascular pressure at each arterial node. The simulation connects three time-dependent processes: tubular flow and pressure, vascular flow and pressure, and the vascular electrical process, and simulates both the irregular tubular pressure fluctuations that have been observed experimentally (Holstein-Rathlou and Leyssac, 1986; Yip et al., 1992; 1993; Holstein-Rathlou, 1987; Holstein-Rathlou and Marsh, 1989), and autoregulation of blood flow by the entire cluster. The tubular flow fluctuations in the simulations are consistent with result of laser speckle contrast imaging (Holstein-Rathlou et al., 2011; Brazhe et al., 2014; Mitrou et al., 2015; Postnov et al., 2022.), and have also been recorded in conscious humans using functional magnetic resonance imaging (Baldelomar et al., 2024). The simulations were limited to time periods of approximately 1 h, the length of time covered in experimental results used to validate the model results.

The largest Lyapunov exponent is used to assess the dynamical state of systems, its sign indicating the nature of the dynamics (Kantz and Schreiber, 1998; Pikovsky et al., 2001; Osipov et al., 2010). A positive value reflects exponential divergence of trajectories in time, a characteristic of chaotic dynamics. A negative value reveals exponential convergence towards a fixed point, and a value of 0 shows the presence of a stable limit cycle (regular oscillation) or a stable flow on a torus (quasi-periodicity). Convergence of successive trajectories in a system is a property that leads to insensitivity to initial conditions; diversion of successive trajectories is the opposite and leads to sensitivity to initial conditions, and in general the associated attractor will explore a larger region of the phase space compared to attractors characterized by largest Lyapunov exponents that are 0 or negative.

The largest Lyapunov exponents measured in 10 nephrons at seven different root blood pressures were positive, indicating that all nephrons were operating with chaotic dynamics. Because each of the 10 nephrons were provided with a parameter set that produces limit cycle oscillations in one - and two-nephron versions, the emergence of chaotic dynamics must be due to the differences in the environment and in the interactions the network creates. One environmental difference is that the electrical current flows from the smooth muscle to the ground potential (0 V) in the one- and two-nephron models, and to an endothelial cell with a variable potential in the network model. The interactions in the network model are among the tubular model controlled by TGF, the endothelial cell electrical potential receiving currents from both the afferent arteriole immediately adjacent and propagated over the network from the other nephrons, and from the fluctuations in vascular pressure induced by time-varying withdrawal in other nephrons. Dynamical systems can bifurcate from limit cycle oscillation to chaotic dynamics when phase coupling changes or when amplitude of the attractor increases (Pikovsky et al., 2001). The nephron vascular network invokes both of these mechanisms, as illustrated in Figure 3. The response of the nodal electrical potential with increasing blood pressure was to change the time interval between myogenic oscillations, in effect changing the phase angle between them. Increasing blood pressure increases the TGF signal strength (Marsh and Holstein-Rathlou, 2024), and the TGF signal modulates the amplitude and frequency of the myogenic oscillation (Marsh et al., 2005a; Marsh et al., 2005b). Thus, interactions among various signals induce chaotic dynamics.

Measurements in anatomic studies show topological variation, and there is also variation in arterial lengths (Postnov et al., 2016; Marsh et al., 2017). To test whether the results were altered by topologic or length variations, we constructed two variations on the original model structure (see Figure 5). The Lyapunov exponent results showed minor differences, but all Lyapunov estimates were positive, and showed similar variation when arterial blood pressure was increased. The lack of change with structure is consistent with the finding that the effect of electrical signaling was more powerful than the effect of arterial pressure signaling.

The anatomical features of the renal arterial network provide a topology that supports the development of chaotic dynamics. The arterial origins of afferent arterioles are most often paired, and less often singular (Marsh et al., 2017). The paired arterioles share a vascular node and therefore experience identical endothelial cell electrical potentials, leading to complete synchronization, even when their arterial lengths differ (Marsh et al., 2023). Single origin afferent arterioles synchronize only partially with paired afferent arterioles or with other adjacent single afferent arterioles because of the electrical resistance provided by the endothelial distance separating them (Marsh et al., 2023). This lack of complete synchronization should lead to a cluster of arterioles with non-identical time series, a theoretical prescription that also corresponds to a description of experimental results (Holstein-Rathlou and Leyssac, 1986; Yip et al., 1992; 1993; Holstein-Rathlou, 1987; Holstein-Rathlou and Marsh, 1989; Holstein-Rathlou et al., 2011; Brazhe et al., 2014; Mitrou et al., 2015; Postnov et al., 2022.).

Although all calculated Lyapunov exponents were positive, their magnitudes were close to 0. This indicates that the degree of chaos in the system is low, as is also evident from the time series and the phase portraits in Figures 3, 6. As a further test for the presence of chaos in the system, we tested the predictability of the system by imposing a small perturbation in tubular pressure, and compared the time evolution of the perturbed system to that of the reference. A chaotic system is characterized by phase space divergence of the trajectories, and as consequence will have limited predictability (Eckmann et al., 1986; Sano and Sawada, 1985). The results shown in the bottom panels of Figure 6 confirm that the attractors had a limited prediction time, and that the magnitude of the prediction horizon scaled with the largest Lyapunov exponent as expected for a chaotic system.

Previous work has suggested that adaptive networks evolve at “the-edge-of-chaos”, and that this provides optimal conditions for information transmission, storage, and modification (Langton, 1990; Sprott, 2008). Strong chaos, as seen in many simple systems like the Lorenz and the Rössler systems, would result in loss of system structure, utility and adaptability (Sprott, 2008). A weakly chaotic system retains some regularity, but at the same time visits a larger volume of the state space providing optimal conditions for adaptive responses. We speculate that had nephron dynamics remained those of limit cycle oscillators, the insensitivity to initial conditions together with the limited volume of phase space visited by the attractor, would have restricted the adaptive capacity needed to sustain autoregulation (Shinbrot et al., 1993).

To assess the quantitative importance of each interaction we calculated the mutual information between each interaction pair in each of the 10 nephrons at 7 different node 11 arterial pressures. The interacting physical processes represented in the model do not share a common dimension, can have negative or positive values in those dimensions, and are generated by non-linear systems. Information calculations are based on probability estimates, have units of bits, and are indifferent to whether the signals originate from linear or non-linear sources. Information streams can be discrete or continuous; those in our model originate from processes described by differential equations and are therefore continuous, giving them units of bits/sec. The information calculation generates a probability density function, and the calculation of mutual information provides a quantitative measure of the overlap of the probability density functions. The results in Figures 7, 8 show that interaction between tubular pressure and nodal electrical potential is more frequently stronger than the interaction between tubular pressure and artery nodal blood pressure. Of the 70 values in each of the tables, 55 are higher for the tubular pressure-nodal PD interaction. Tubular pressure dynamics develop under control of afferent arterioles, whose dynamics requires changes in membrane electrical potential differences. The dominance of the tubular pressure - nodal PD interaction is consistent with this formulation.

Neither of the 2 figures reveal discernible trends in the dominance of one pair of interactions over the other, either with respect to nephron number or root arterial blood pressure. The chaotic dynamics of the various processes and the asymmetries of the system’s structure provide a likely explanation. We suggest that the major achievement of this system is that its emergent property is the formation of a functional nephron cluster capable of exchanging information among its members, its members capable of responding to the information exchange, and allowing the system to achieve autoregulation.

The model was developed assuming a constant arterial blood pressure at the root of the network and was exercised with a step increase in root pressure to a higher level that was held constant during the analyzes. As noted above, the arterial blood pressure of a conscious mammal does not remain constant with time but exhibits *1/f* characteristics over epochs similar to those used in the simulation. When challenged with a *1/f* time varying root pressure the model continued to sustain autoregulation, and a spectral peak at the TGF frequency. We conclude that the model accounts for renal stability for periods of at least 1 h and, in doing so, provides a clock function that can serve to synchronize a variety of tubular functions.

We end by noting, as we have previously (Marsh et al., 2023), that the experimental results used to validate the model’s predictions were all collected from the ventral surface of the kidneys of anesthetized young male rats, during the time of day when the rats were normally least active. The structural measurements were taken from the same region of the kidney (Marsh et al., 2017). The structure of the present model, while consistent with the structural data, may or may not exist in all details in different regions of the kidney. The rat kidney contains more than 30,000 nephrons, so the space of possible topologies is very large. Furthermore, the nephrons modelled are surface nephrons having loops of Henle that are restricted to the outer medulla. Juxtamedullary nephrons entering the inner medulla have variable lengths, all longer than those restricted to the outer medulla. We caution that the dynamics we observed and modeled are from a restricted region of the kidney, for a limited number of nephrons, and during a limited time period. The results should therefore be viewed as illustrative of the consequences of the interactions on the network, and not necessarily a complete characterization.

## Data Availability

The raw data supporting the conclusions of this article will be made available by the authors, without undue reservation.

## References

[B1] Baldelomar E. J. Morozov D. Wilson L. D. Eldeniz C. An H. Charlton J. R. (2024). Resting-state mri reveals spontaneous physiological fluctuations in the kidneyand tracks diabetic nephropathy in rats. Am. J. Physiol. Ren. Physiol. 327, F113–F127. 10.1152/ajprenal.00423.2023 38660712 PMC11390131

[B2] Blinowska K. Marsh D. J. (1985). Ultra- and circadian fluctuations in arterial pressure and electromyogram in conscious dogs. Am. J. Physiol. Regul. Integr. Comp. Physiol. 249, R720–R725. 10.1152/ajpregu.1985.249.6.R720 4073292

[B3] Brazhe A. Marsh D. J. Holstein-Rathlou N.-H. Sosnovtseva O. (2014). Synchronized renal blood flow dynamics mapped with wavelet analysis of laser speckle flowmetry data. PLoS ONE 9, e105879. 10.1371/journal.pone.0105879 25216274 PMC4162534

[B4] Chou C. L. Marsh D. J. (1988). Role of proximal convoluted tubule in pressure diuresis in the rat. Am. J. Physiol. Ren. Physiol. 254, F601–F607. 10.1152/ajprenal.1988.254.4.F601 3740275

[B5] Chou C. L. Marsh D. J. (1998). Time course of proximal tubule response to acute arterial hypertension in the rat. Am. J. Physiol. Ren. Physiol. 275, F565–F575. 10.1152/ajprenal.1988.254.4.F601 3354689

[B6] Eckmann J.-P. Ruelle D. (1985). Ergodic theory of chaos and strange attractors. Rev. Mod. Phys. 57, 617–656. 10.1103/RevModPhys.57.617

[B7] Eckmann J.-P. Kamphorst S. O. Ruelle D. Ciliberto S. (1986). Liapunov exponents from time series. Phys. Rev. A 34, 4971–4979. 10.1103/physreva.34.4971 9897880

[B8] Edwards A. McDonough A. A. Ellison D. H. (2025). Flow-dependent transport processes 2024: filtration, absorption, and secretion. Am. J. Physiol. Ren. Physiol. 328, F627–F637. 10.1152/ajprenal.00303.2024 40094947 PMC12053844

[B9] Gonzalez-Fernandez J. M. Ermentrout G. B. (1994). On the origin and dynamics of the vasomotion of small arteries. Math. Biosci. 240, 127–167. 10.1016/0025-5564(94)90074-4 8142694

[B10] Holstein-Rathlou N.-H. (1987). Synchronization of proximal intratubular pressure oscillations: evidence for interaction between nephrons. Pfluegers Arch. 408, 438–443. 10.1007/BF00585066 3601634

[B11] Holstein-Rathlou N.-H. Leyssac P. P. (1986). TGF-mediated oscillations in the proximal intratubular pressure: differences between spontaneously hypertensive rats and wistar-kyoto rats. Acta Physiol. Scand. 126, 333–339. 10.1111/j.1748-1716.1986.tb07824.x 3962682

[B12] Holstein-Rathlou N.-H. Marsh D. J. (1989). Oscillations of tubular pressure, flow, and distal chloride concentration in rats. Am. J. Physiol. Ren. Physiol. 256, F1007–F1014. 10.1152/ajprenal.1989.256.6.F1007 2735418

[B13] Holstein-Rathlou N.-H. He J. Wagner A. Marsh D. J. (1995). Patterns of blood pressure variability in normotensive and hypertensive rats. Am. J. Physiol. Reg. Int. Comp. Physiol. 269, R1230–R1239. 10.1152/ajpregu.1995.269.5.R1230 7503315

[B14] Holstein-Rathlou N.-H. Sosnovtseva O. V. Pavlov A. N. Cupples W. A. Sorensen C. M. Marsh D. J. (2011). Nephron blood flow dynamics measured by laser speckle contrast imaging. Am. J. Physiol. Ren. Physiol. 300, F319–F329. 10.1152/ajprenal.00417.2010 21048025 PMC3044006

[B15] Kantz H. Schreiber (1998). Nonlinear Time Series Analysis. Cambridge, United Kingdom: Cambridge University Press.

[B16] Krakauer D. C. (2023). Symmetry–simplicity, broken symmetry–complexity. Interface Focus 13, 20220075. 10.1098/rsfs.2022.0075 37065260 PMC10102721

[B17] Langton C. G. (1990). Computation at the edge of chaos: phase transitions and emergent computation. Phys. D. 42, 12–37. 10.1016/0167-2789(90)90064-v

[B18] Laugesen J. Sosnovtseva O. Mosekilde E. Holstein-Rathlou N.-H. Marsh D. J. (2010). Coupling-induced complexity in nephron models of renal blood flow regulation. Am. J. Physiol.Regul. Integr. Comp. Physiol. 298, R997–R1006. 10.1152/ajpregu.00714.2009 20147606 PMC2853389

[B19] Marsh D. J. Holstein-Rathlou N.-H. (2024). Response of the nephron arterial network and its interactions to acute hypertension: a simulation. Function 6, zqae049. 10.1093/function/zqae049 39533815 PMC11931624

[B20] Marsh D. J. Osborn J. Cowley A. (1990). *1/f* fluctuations in arterial pressure and regulation of renal blood flow in dogs. Am. J. Physiol. Ren. Physiol. 258, F1394–F1400. 10.1152/ajprenal.1990.258.5.F1394 2337155

[B21] Marsh D. J. Sosnovtseva O. Chon K. Holstein-Rathlou N.-H. (2005a). Nonlinear interactions in renal blood flow regulation. Am. J. Physiol. Regul. Integr. Comp. Physiol. 288, R1143–R1159. 10.1152/ajpregu.00539.2004 15677526

[B22] Marsh D. J. Sosnovtseva O. Pavlov A. Yip K. Holstein-Rathlou N.-H. (2005b). Frequency encoding in renal blood flow regulation. Am. J. Physiol. Regul. Integr. Comp. Physiol. 288, R1160–R1167. 10.1152/ajpregu.00540.2004 15661968

[B23] Marsh D. J. Toma I. Sosnovtseva O. Peti-Peterdi J. Holstein-Rathlou N.-H. (2009). Electrotonic vascular signal conduction and nephron synchronization. Am. J. Physiol. Ren. Physiol. 296, F751–F761. 10.1152/ajprenal.90669.2008 19116241 PMC2670644

[B24] Marsh D. J. Postnov D. Rowland D. Wexler A. Sosnovtseva O. Holstein-Rathlou N.-H. (2017). Architecture of the rat nephron-arterial network: analysis with micro computed tomography. Am. J. Physiol. Ren. Physiol. 313, F351–F360. 10.1152/ajprenal.00092.2017 28424208 PMC5582900

[B25] Marsh D. J. Wexler A. S. Holstein-Rathlou N.-H. (2023). Interacting information streams on the nephron arterial network. Front. Netw. Physiology 3, 1254964. 10.3389/fnetp.2023.1254964 37928058 PMC10620968

[B26] Mitrou N. Scully C. Braam B. Chon K. Cupples W. A. (2015). Laser speckle contrast imaging reveals large-scale synchronization of cortical autoregulation dynamics influenced by nitric oxide. Am. J. Physiol. Ren. Physiol. 308, F661–F670. 10.1152/ajprenal.00022.2014 25587114

[B27] Osipov G. V. Kuerths J. Zhou C. (2010). Synchronization in Oscillatory Networks. Berlin Heidelberg: Springer-Verlag.

[B28] Pikovsky A. Rosenblum M. Kuerths J. (2001). Synchronization. Cambridge, United Kindom: Cambridge University Press.

[B29] Postnov D. Marsh D. J. Postnov E. Braunstein T. Holstein-Rathlou N.-H. Martens E. (2016). Modeling of kidney hemodynamics: probability-based topology of an arterial network. PLoS Comput. Biol. 12, e1004922. 10.1371/journal.pcbi.1004922 27447287 PMC4957782

[B30] Postnov D. Marsh D. J. Cupples W. A. Holstein-Rathlou N.-H. Sosnovtseva O. V. (2022). Synchronization in renal microcirculation unveiled with high resolution blood flow imaging. eLife 11, e75284. 10.7554/elife.75284 35522041 PMC9113743

[B31] Sano M. Sawada Y. (1985). Measurement of the lyapunov spectrum from a chaotic time series. Phys. Rev. Lett. 55, 1082–1085. 10.1103/PhysRevLett.55.1082 10031723

[B32] Shannon C. E. Weaver W. (1998). The Mathematical Theory of Communication. Urbana, Ill: University of Illinois Press.

[B33] Shinbrot T. Grebogi C. Yorke J. A. Ott E. (1993). Using small perturbations to control chaos. Nature 363, 411–417. 10.1038/363411a0

[B34] Sorensen C. M. Leyssac P. P. Skott O. Holstein-Rathlou N.-H. (2000). Role of the renin-angiotensin system in regulation and autoregulation of renal blood flow. Am. J. Physiol. Reg. Int. Comp. Physiol. 79, R1017–R1024. 10.1152/ajpregu.2000.279.3.R1017 10956261

[B35] Sorensen C. M. Leyssac P. P. Salomonsson M. Skott O. Holstein-Rathlou N.-H. (2004). Ang ii-induced downregulation of RBF after a prolonged reduction of renal perfusion pressure is due to pre- and postglomerular constriction. Am. J. Physiol. Reg. Int. Comp. Physiol. 86, R865–R873. 10.1152/ajpregu.00424.2003 14715487

[B36] Sosnovtseva O. Pavlov A. Mosekilde E. Yip K. Holstein-Rathlou N.-H. Marsh D. J. (2007). Synchronization among mechanisms of renal autoregulation is reduced in hypertensive rats. Am. J. Physiol. Ren. Physiol. 293, F1545–F1555. 10.1152/ajprenal.00054.2007 17728377

[B37] Sprott J. C. (2008). Chaotic dynamics in large networks. Chaos 18, 023135. 10.1063/1.2945229 18601501

[B38] Wagner C. Persson P. (1994). Two ranges in blood pressure power spectrum with different 1/f characteristics. Am. J. Physiol. Heart Circ. Physiol. 267, H449–H454. 10.1152/ajpheart.1994.267.2.H449 7915083

[B39] Wagner A. J. Holstein-Rathlou N.-H. Marsh D. J. (1997). Internephron coupling by conducted vasomotor responses in normotensive and spontaneously hypertensive rats. Am. J. Physiol. Ren. Physiol. 272, F372–F379. 10.1152/ajprenal.1997.272.3.F372 9087681

[B40] Yip K. Holstein-Rathlou N.-H. Marsh D. J. (1992). Dynamics of TGF-initiated nephron-nephron interactions in normotensive rats and SHR. Am. J. Physiol. Ren. Physiol. 262, F980–F988. 10.1152/ajprenal.1992.262.6.F980 1621821

[B41] Yip K. Holstein-Rathlou N.-H. Marsh D. J. (1993). Mechanisms of temporal variation in single-nephron blood flow in rats. Am. J. Physiol. Ren. Physiol. 264, F427–F434. 10.1152/ajprenal.1993.264.3.F427 8456956

